# Dense Convolutional Network and Its Application in Medical Image Analysis

**DOI:** 10.1155/2022/2384830

**Published:** 2022-04-25

**Authors:** Tao Zhou, XinYu Ye, HuiLing Lu, Xiaomin Zheng, Shi Qiu, YunCan Liu

**Affiliations:** ^1^School of Computer Science and Engineering, North Minzu University, Yinchuan 750021, China; ^2^Key Laboratory of Image & Graphics Intelligent Processing of State Ethnic Affairs Commission, North Minzu University, Yinchuan 750021, China; ^3^School of Science, Ningxia Medical University, Yinchuan 750004, China; ^4^Research Institute for Reproductive Medicine and Genetic Diseases, Wuxi Maternity and Child Health Hospital, Wuxi, Jiangsu 214002, China; ^5^Xi'an Institute of Optics and Precision Mechanics, Chinese Academy of Sciences, Xi'an 710119, China

## Abstract

Dense convolutional network (DenseNet) is a hot topic in deep learning research in recent years, which has good applications in medical image analysis. In this paper, DenseNet is summarized from the following aspects. First, the basic principle of DenseNet is introduced; second, the development of DenseNet is summarized and analyzed from five aspects: broaden DenseNet structure, lightweight DenseNet structure, dense unit, dense connection mode, and attention mechanism; finally, the application research of DenseNet in the field of medical image analysis is summarized from three aspects: pattern recognition, image segmentation, and object detection. The network structures of DenseNet are systematically summarized in this paper, which has certain positive significance for the research and development of DenseNet.

## 1. Introduction

Deep learning is an end-to-end approach to extracting and abstracting image features layer by layer and implementing recognition functions. Krizhevsky et al. [1] used deep learning to win the champion of computer vision challenges in ImageNet. Therefore, deep learning methods represented by convolutional neural networks have become a popular research topic in the field of pattern recognition. GoogLeNet, ResNet, and DenseNet have been proposed one after another. Among them, DenseNet [2] has achieved better results among many deep learning models due to the new architecture of dense connectivity, DenseNet achieves interconnection between arbitrary layers and skip connection mode that transfers information from shallow layers directly to deep layers, and enhanced feature transfer and feature reuse between network layers, resulting in a more compact network representation with less feature redundancy. Under the condition of same layer depth, the convergence performance of network is better, the network degradation and gradient disappearance problems caused by the deepening of convolutional network are better alleviated, and the number of network parameters and computational efficiency are significantly reduced.

Litjens et al. [3] point out that deep learning algorithms, especially convolutional networks, have been successful in medical image analysis. Xie et al. [4] point out that deep learning is widely used in medicine for disease diagnosis, lesion and abnormality detection, lesion and organ segmentation, etc. In recent years, deep learning has developed rapidly in the medical field, represented by companies such as Watson, Microsoft Nuance, Google Health in the world and iFlytek, Tencent Miying, and Ali Health in China. Eagle Pupil Airdoc [5] fundus image-assisted diagnosis system for diabetic retinopathy (DR) has high sensitivity and specificity in detecting, DR. Dr. Wise [6], launched by DeepMed, can achieve rapid identification, accurate measurement, diagnostic analysis, and scientific follow-up of pneumonia signs, providing clinicians with a basis for treatment, thus realizing precision medicine.

DenseNet has made a breakthrough in medical image analysis tasks [7]; Li and Liu [8] used DenseNet to learn local block features of MRI brain image clusters and achieved a better Alzheimer's disease classification accuracy of 89.5%; Jégou et al. [9] extended densely connected structure to fully convolutional neural network by introducing different depth paths to produce different scale nonlinear mappings for semantic segmentation, thus avoiding gradient disappearance and enabling deeper networks to be trained with fewer parameters; Ke et al. [10] based on 3D DenseNet performed automatic detection and segmentation of nasopharyngeal carcinoma in MRI images, which can obtain higher overall accuracy, sensitivity, and specificity than radiologists. DenseNet can provide clinical aid diagnostic solutions for major diseases such as benign and malignant tumors, cardiovascular and cerebrovascular diseases, and respiratory diseases. Liu et al. [11] based on DenseNet extracted spatiotemporal features of patients' cardiac magnetic resonance imaging (MRI) to predict the ray fraction of left ventricle; Kishan and Janardhan [12] aimed at the problem of small intracerebral hemorrhage and high square difference and used a combination of DenseNet and Inception V3 models to accurately detect and identify early intracranial hemorrhage; Li et al. [13] used transfer learning and DenseNet to classify benign and malignant COVID-19 CT images, reaching the state-of-the-art in terms of accuracy and F1 score.

This paper summarizes DenseNets, and the rest of the structure is as follows: Section 2 introduces the basic principles of DenseNet; Section 3 reviews the development of DenseNet models and summarizes five aspects of broaden network structure, lightweight network structure, dense unit, dense connection mode, and attention mechanism; Section 4 summarizes the application of DenseNet in field of medical image analysis and explores three aspects of pattern recognition, image segmentation, and object detection.

## 2. Basic Principles of DenseNet

**Figure 1:**
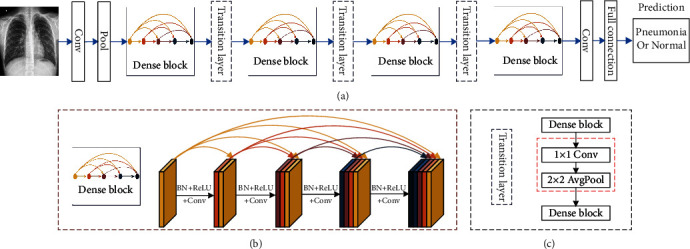
DenseNet structure. (a) The basic structure of DenseNet consists of dense block, transition layer, convolutional layer, and fully connected layer. (b) Denseblock consists of densely connected dense units with nonlinear mapping functions of BN, ReLU, and Conv, which are designed with preactivation strategy to make network training easier and generalization performance better. Dense unit input is spliced and merged with all outputs of the previous dense units, and new features generated also need to be passed to subsequent dense units, so that shallow features of dense block are repeatedly reused and effectively utilized, which can alleviate gradient disappearance to a certain extent, and a large number of features can be generated with a small number of convolution kernels; final DenseNet model is relaticely in scale. (c) Transition layer is the structure between adjacent dense blocks, which consists of 1 × 1 convolution and 2 × 2 average pooling layer, compressing dense block input and all extracted feature information, reducing feature map size and dimensionality, which can effectively reduce the number of dense block parameters and prevent network from overfitting. The fully connected layer is classification prediction layer, which reduces the influence of feature location on classification by integrating category feature information in network features, and classifies feature information after weighting.

In 2017, Huang et al. [2] proposed DenseNet, a convolutional neural network with densely connected structure, and DenseNet structure is shown in Figure 1.

DenseNet has the characteristics of feature sharing and arbitrary interlayer interconnection. The advantages of DenseNet are feature map reuse through dense connection, reducing interdependence between layers by reusing feature maps from different layers, providing compact and differentiated input features by shortcut connections of different lengths, and effectively reducing the gradient disappearance problem that is difficult to optimize in deep networks. The final prediction is to use features from all layer to obtain better performance and model robustness on a standard dataset with smaller model size and computational effort. The disadvantage of DenseNet is that the feature maps of each layer are spliced with the previous layer, and the data is replicated multiple times. As the number of network layers increases, the number of model parameters grows linearly, eventually leading to explosive growth in computation and memory overhead during training.

## 3. Development of DenseNet

**Figure 2:**
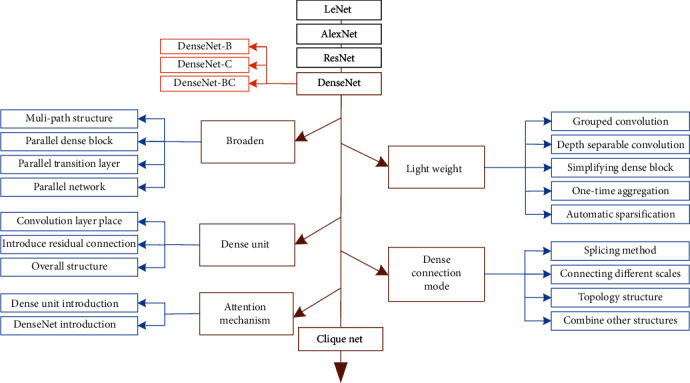
Development of DenseNet.

There are five improvement methods of DenseNet, such as broaden DenseNet structure, lightweight DenseNet structure, dense unit, dense connection mode, and attention mechanism. This section summarizes the development of DenseNet, as shown in Figure 2.

In 1998, LeCun et al. [14] proposed LeNet model, in which some new operators such as convolutional operator, pooling operator, and full connection operator were used, and LeNet become a pioneer model in deep learning models.

In 2012, Krizhevsky et al. [1] proposed AlexNet, in which ReLU was used for activation function; network architecture uses dual GPUs, reduced pooling steps, enhanced training data, and dropout function to avoid overfitting; this network achieved 10.9% better result than second place in ILSVRC competition.

In 2015, He et al. [15] proposed ResNet for the problem of network with more stacked structural layers but fast performance degradation, using residual connections to transfer shallow features directly to deeper layers, so that the originally fitted output *H*(*x*) becomes the residual output *H*(*x*) − *x*. Even when learning structural layer with 0 features, ResNet only does constant mapping and learns new features based on input features; ResNet learns new features based on input features, alleviating the degradation problem that occurs in deep models and giving network better performance.

In 2017, Huang et al. [2] further improved feature reuse capability based on ResNet and proposed DenseNet network with dense connection operation, but in the case of dense connection as the number of layers of dense blocks increases, input feature map dimension at the proximal end becomes larger and larger, to alleviate this problem Huang et al. [2] added 1 × 1 convolution (bottleneck unit) into dense unit to reduce the dimension of input feature maps, reducing the computational effort while incorporating different dense unit features. DenseNet-B uses 1 × 1 convolution to reduce input feature map dimension of each layer to a fixed multiple of growth rate; DenseNet-C adds compression rate in transition layer to reduce output feature dimension of dense blocks; DenseNet-BC adds both bottleneck unit and variable compression rate transition layer.

Broaden DenseNet structure is multibranch improvement of network single layer, network structure block, and whole network using multibranch extraction function, including multipath structure, parallel dense block, parallel transition layer, and parallel network.

Lightweight DenseNet structure significantly reduces the number of network parameters and computation from two aspects: network model architecture design and network model compression. Network model architecture design which includes grouped convolution, depth-separable convolution, simplified dense block, one-time aggregation, and network model compression has automatic sparseness.

Dense unit is composite function improvement of DenseNet, optimizing feature extraction module dense unit for specific applications, summarized in three aspects: convolution layer placement, introduce residual connection, and overall structure.

Dense connection mode is a study of the way dense blocks are composed, dense connection improvement has four aspects: splicing method, connect dense unit of different scales, topological structure, and combine with other structure.

Attention mechanism is to emphasize or select important features in DenseNet and suppress some irrelevant detailed information, which can be divided into attention mechanism in dense unit and DenseNet.

Chen et al. [16] indicated that densely connected path of DenseNet increases linearly with increasing network depth, and the parameters increase dramatically; for this reason, Yang et al. [17] proposed CliqueNet, a convolutional neural network with alternating update mechanism, Clique block performs same dense propagation as DenseNet, and then, all layers except the top layer are used as the bottom layer for alternating updates, stitching feature maps of each layer into the output of Clique block, passing only fixed-dimensional feature maps, with forward and backward connections between any layer, cycling, and alternating updates to maximize the flow of information between layers. Final network uses fewer convolutional kernels to generate a large number of features, which greatly improves the parameter efficiency, and parameter propagation process generates multilevel features by cycling; experiments show that the second stage of Clique block helps to suppress noise and improve performance.

### 3.1. Broaden DenseNet Structure

Depth is not necessarily good, and width is not necessarily bad. On the one hand, in 2014, Ba and Caruana [18] pointed out that a well-trained deep network can usually find a suitable shallow network instead, and in 2019, Hanin and Rolnick [19] pointed out that expressiveness of network in practical applications is independent of network depth and only grows linearly with the number of neurons; on the other hand, deep learning models currently are trained by GPUs. GPU parallel processing makes it easier to train widened networks. Therefore, depth and width are not completely opposed to each other, increasing both depth and width is increasing the number of learnable parameters and improving network fitting ability, increasing depth can obtain a larger sensory field and capture more pixel-like features, and increasing width can obtain more subtle and richer features.

**Figure 3:**
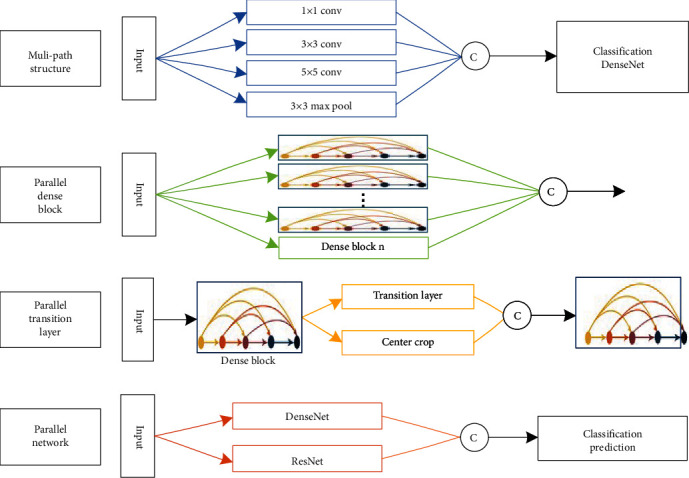
Broaden DenseNet structure.

Broaden DenseNet structure is using multiple branches to improve network single layer, structure block, and whole, so that network learns more rich features per layer, such as texture features with different directions and frequencies. Although network deepening can extract knowledge layer by layer abstraction and continuous refinement, too narrow network can capture limited patterns per layer, and the network is no deeper to extract information. Broaden DenseNet structure approach is shown in Figure 3; there are multipath structure, parallel dense block, parallel transition layer, and parallel network.

First, multipath structure is an improvement of multipath feature extraction layer on single-path feature extraction, using multiple scales to achieve different perceptual fields, increasing the adaptability of network to features of different sizes and enriching features. Multipath convolution also uses the computational principle of decomposing sparse matrices into dense matrices to speed up convergence, aggregating output features with strong relevance, decomposing feature sets into multiple densely distributed subsets, aggregating features with strong relevance, and finally outputting less redundant information and faster convergence. In 2014, Google's Szegedy et al. [20] proposed typical multipath structure inception, which is parallel to commonly used 1 × 1, 3 × 3, 5 × 5 convolutions and 3 × 3 max-pooling to achieve different perceptual fields for extracting rich features and making classification judgments more accurate. Li et al. [21] proposed DenseNet-II for benign and malignant classification of mammogram images; the inception structure was used to improve adaptability of network to different scales and maintain the sparsity of network structure, while efficiently expanding network depth and width, resulting in better classification performance with good generalization and robustness. Arega and Bricq [22] used cascaded segmentation network FC-DenseNet for automatic segmentation of cardiac scars in multisequence cardiac MRI; similar inception structure captures relevant features such as scars and edema of different sizes, significantly improving segmentation results with the same computational overhead.

Second, parallel dense block is original single-branch dense block multiple times in parallel to increase network width, combining the benefits of network depth and width to improve model robustness; although deep networks have higher performance, they are limited in terms of gradient disappearance, reduced forward flow, and slower training time. Lodhi and Kang [23] proposed multipath-DenseNet with multiple dense blocks parallel learning to find valid paths from some or all of the blocks, which allows efficient gradient flow and achieves higher accuracy than other baselines using fewer parameters on the classification dataset. Chen et al. [24] proposed MFR-DenseNet with multipath feature recalibration, where SE module assembles multipath dense blocks from different channel feature maps to build interchannel feature correlation model to achieve strong feature extraction, which achieves high accuracy in classification while maintaining training stability.

Third, parallel transition layer is a branching supplement based on the single-branch transition layer to realize broadening. Liu et al. [25] proposed dense binary tree DenseBTNet for lung nodule classification, which introduces central cropping operation into transition layer and parallelizes it to create indirect shortcuts between different dense blocks, preserving detail features of nodule morphology and filtering irrelevant background information; binary tree-like singular network model can generate rich multiscale features, making the parameter scale lighter and with higher parameter efficiency.

Fourth, parallel network is multiple networks in parallel, and the fusion of multiple network features can ensure more stable and accurate prediction. Chen et al. [16] argued that ResNet is essentially DenseNet with shared connections, and ResNet implicitly has residual path reuse features, and dense connections that can be mined for new features, based on this joint perspective dual-channel network (DPN) were proposed, by sharing same features of network through dual-path structure; high accuracy is achieved with flexibility, small models, less computation, and low resources. Nguyen et al. [26] proposed MSDENSE-DAT to predict regions in crowd scenes that attract human attention, designed two-branch homography network based on DenseNet201 to extract multiscale features, extracted as many features as possible from original size and half-size images, and cascaded two-branch features along channel axis, and also designed self-attention blocks to emphasize interfeature correlation; MSDENSE-DAT extracts the best features with low density in the crowd.

### 3.2. Lightweight DenseNet Structure

**Figure 4:**
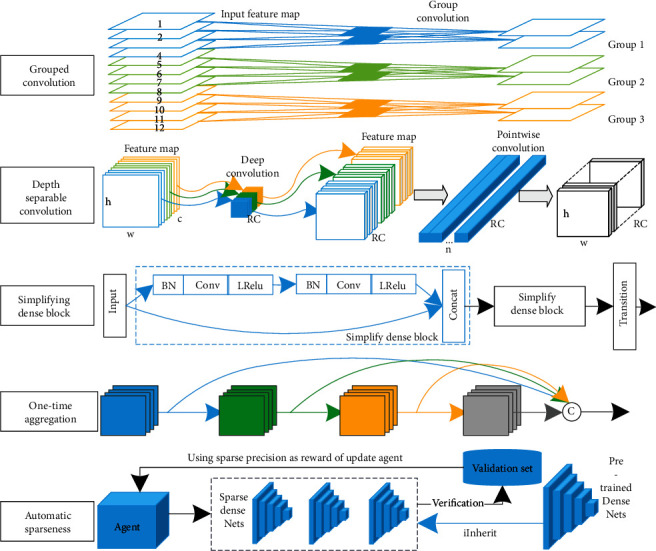
Lightweight DenseNet structure.

Lightweight of CNN is to reduce the number of parameters and computation without degrading the performance of existing models and to achieve storage and computation on mobile platforms with limited storage space and power consumption. CNN is widely used in image analysis and has achieved great success, greatly reducing the number of parameters and computation through weight sharing and sparse connection; however, two aspects, model storage and model prediction speed, limit the application of CNN in mobile. Lightweight of CNN is important research in deep learning, and two main directions are studied: lightweight model architecture design and model compression. This section summarizes four improvements of lightweight DenseNet structure in model architecture design: grouped convolution, depth separable convolution, simplified dense block and one-time aggregation, and automatic sparseness of model compression, as shown in Figure 4.

First, grouped convolution is to group feature maps and then convolve them separately, the number of model parameters decreases as the number of groups decrease, and model structure and training are more efficient. In 2012, AlexNet [1] proposed grouped convolution and utilized dual GPU processing. To apply DenseNet to mobile devices with limited computational resources, Huang et al. [27] proposed CondenseNet based on grouped convolution, where the network is connected layer by layer, preserving dense connectivity of DenseNet, achieving feature reuse, sharing input features using self-learning grouped convolution modules, eliminating layer-to-layer connections, and avoiding accuracy branching process that affects too much; CondenseNet uses only 1/10th of computation at an accuracy level comparable to that of DenseNet. Li et al. [28] proposed Dense2Net with efficient multilevel group convolution, using channel mixing to rearrange features, tiling features into multiple groups and randomly receiving the previous group of features, while fusing all groups of different scales into output using 1 × 1 convolution to improve parameter efficiency and information transfer; Dense2Net has higher accuracy and better information transfer than DenseNet.

Second, depth separable convolution (DWSC) is a decomposition of the convolution strategy into spatial convolution followed by channel convolution, replacing convolution layer to achieve a lightweight network, using a filter for each input channel in spatial directional convolution (DWC), and applying 1 × 1 point-by-point convolution (PWC) on the channel side to linearly combine the depth directional convolution outputs. MobileNet [29] uses depth separable convolution to build a lightweight deep network that greatly reduces the computational effort with a small reduction in accuracy, achieves a balance between model efficiency and classification accuracy, and performs well in task such as target detection, fine classification, and face attribute. Chen et al. [30] proposed Mobile-DANet, an attention-embedded lightweight network for corn disease recognition, using depth-separable convolution instead of convolutional layers in dense blocks and depth convolution using input mapping filters to achieve channel-by-channel convolution and combining channel-by-channel results through 1 × 1 convolution, while using spatial and channel attention mechanism to detect and highlight targets in multifeature map, with the final model featuring small size, high accuracy, and excellent performance.

Third, simplified dense block is to replace original dense block with multiple simplified dense blocks in series, fixing the number of dense units to be smaller and multiple simplified dense blocks in series to replace original dense blocks, reducing output feature map dimension while maintaining feature reuse. s-DenseNet was proposed by Yu et al. [31] for pathological grading of breast cancer on large-size mammograms, using fewer dense units to prevent network overfitting, extracting high-level semantic features, and combining logistic regression to select image histology feature values, the trained network achieves high accuracy at lower complexity. Huang et al. [32] designed a lightweight neural network using simplified dense block consisting of two dense units, stitching adjacent blocks of mammogram and osteosarcoma histological images as input; feature vectors were mapped to simplified dense block for kernel learning; final network was easier to train and better in terms of classification accuracy, sensitivity, and specificity.

Fourth, one-time aggregation is to aggregate outputs of all dense units to the end of dense block, rather than two-by-two interconnection of dense units. Lightweight network design considers two main factors, model size and FLOPs, to reduce inference time and energy consumption; memory access cost (MAC) and GPU computational efficiency are also considered. Densely connected aggregations of intermediate features with different sensory fields can produce powerful features using a small number of parameters and triggers, but dense connections can lead to a large memory access overheads, and 1 × 1 convolution using a small number of operations can also lead to inefficient GPU computation. To address the slower and inefficient DenseNet detector, a fast and efficient architecture VoVNet consisting of one-time aggregation (OSA) was proposed by Lee et al. [33], which reduces memory access cost and performs efficient computation by using diverse feature representations with multiple reception rates; dense aggregation is aggregating all previous features to subsequent layers; while OSA is aggregating all features at once to final unit, it also removes bottleneck unit and keeps intermediate layer input constant, greatly improving MAC and GPU efficiency while maintaining cascade strength; VoVNet applied to target detector achieves twice detection speed, and energy consumption is 1.6×–4.1× lower than DenseNet.

Fifth, automatic sparsification method for model compression to achieve lightweight is to prune DenseNet by reinforcement learning. Li et al. [34] proposed automatic sparsification (ADS) method to prune redundant jump connections in DenseNet and ADS compression based on reinforcement learning (RL) method to prune dense connections representation by the original adjacency matrix; sparse DenseNet verification accuracy is used as a reward and punishment for updating RL agents, and high-performance sparse networks are generated by iterating RL agents with fewer parameters, less computation, and portability after sparse.

### 3.3. Dense Unit

DenseNet uses dense cells interconnected two by two to form dense blocks; this section summarizes three aspects of convolution layer placement, and introduces residual connection and overall structure.

#### 3.3.1. Convolution Layer Placement

**Figure 5:**
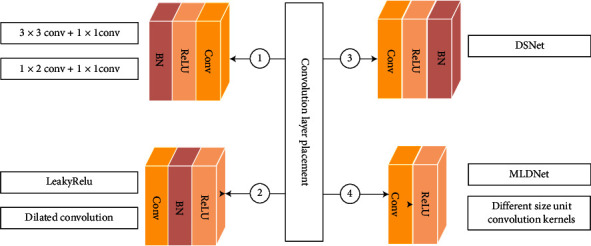
Convolution layer placement.

Dense unit of DenseNet uses preactivation structure, in which BN and ReLU layers achieved preactivation before convolution, and also uses the structure of 1 × 1 convolution followed by 3 × 3 convolution. Dense unit is improved by placement of convolution layer, which can be divided into preactivation and nonpreactivation, where ReLU and BN can interchange their positions, and the specific improved form is shown in Figure 5.

First, convolutional layer is easier to train and has better generalization performance after BN algorithm and preactivation structure of activation function. Zhu et al. [35] achieved image superresolution using dense jump-connected network, where dense unit provides large sensory fields using 3 × 3 convolution, and then, 1 × 1 convolution deepens network to learn robust feature representations, and small filters with a small number of parameters are used to deepen network, making full use of hierarchical feature deepening structure, and network outperforms new algorithm with robust fitting capability. Wei and Liu [36] introduced dense blocks in SSD network to detect hazardous substances in X-ray images. Dense unit connects two 1 × 1 convolutions in series to avoid destruction of learned feature map region information, and the method has good transfer learning capability.

Second, convolutional layer does not use preactivated dense units before BN and activation function. Wei et al. [37] added dense blocks of first convolution in YoloV3 backbone, and transition layer also used dense units in parallel with maximum pooling layer, and the model was effective in multiobjective defect detection of railroad routes. Lei et al. [38] used densely connected dilated convolution for codec connection, and dense blocks were used for first convolution of dilated dense unit, which can enhance effective feature transfer and retain finer structural information for accurate segmentation of skin lesions.

Third, convolutional layer distributes BN-normalized data to the nonsaturated region before activation function and BN, and ReLU can then control the degree of saturation of activation; if the order of BN and ReLU is switched, ReLU will lead to deactivation of some of BN neuron, which causes BN instability and affects the model performance, practically different application scenarios have different role in the order. Hasan et al. [39] proposed an automatic semantic segmentation network (DSnet) for skin lesion segmentation, in which the encoder uses dense blocks and transition layers to avoid learning redundant features and uses BN of dense units after activation function, and DSnet outperforms existing baseline segmentation networks in several metrics.

Fourth, convolutional layer precedes activation function, and BN is more suitable for denoising, but it leads to slow convergence in superresolution and large fluctuation in the loss function, which also increases the memory and computational burden. Mustafa et al. [40] proposed multilevel dense MLDNet for multifocus image fusion (MFIF), which uses parallel dense blocks to extract multilevel local dense and global features in the source image; dense units are connected using convolution followed by ReLU to retain as many dense features as possible, and MLDNet has significant performance improvement over the latest methods. Li et al. [41] proposed multiresolution dense network to connect mutually different structures dense units densely, with different size convolution for feature extraction of three central consistent lung field blocks with different resolutions, and fusion to detect lung nodules, which outperformed general JSRT database for radiologists by up to 99%.

#### 3.3.2. Introduce Residual Connection

**Figure 6:**
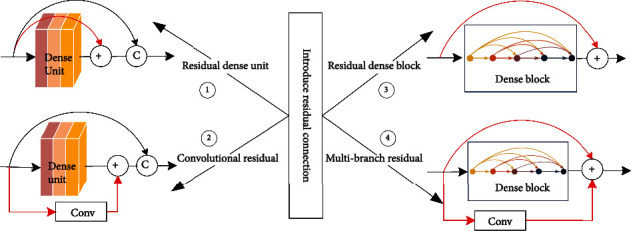
Introduce residual connection.

Residual connection uses skip connection to connect shallow layers of information directly to deep layers, and output is expressed as a linear superposition of input and its nonlinear transformations, using a new expression to achieve a constant mapping between layers. Deep learning relies on error chain derivative back propagation for parameter update; too small derivatives will become smaller and smaller gradients after multiple series multiplications, leading to gradient dispersion; residual connection adds constant terms to derivatives, so that error signal is propagated directly to the shallow layer without intermediate weight matrix changes, which alleviates the problem of gradient dispersion to a certain extent, and information is propagated back and forth more smoothly. Introduction of residual connection in DenseNet can solve network degradation problem, thus deepening network model and improving performance. Four structures are introduced for residual connectivity: residual dense unit, convolutional residual dense unit, residual dense block, and multibranch residual dense block, as shown in Figure 6.

First, residual dense unit is residual connection between inputs and outputs of dense unit, Feng et al. [42] proposed recurrent network DCRN to cope with single-image superresolution (SISR) task, which performs local residual learning within dense block and dense unit to extract semantic features, and experiments on Set5, Set14, BSD100, and Urban100 datasets to demonstrate DCRN efficiency and model component effectiveness.

Second, convolutional residual dense unit is a residual connection using convolutional improvements to prevent gradient disappearance or explosion problems while speeding up convergence. Yang et al. [43] introduced convolutional residual in DenseNet by parallelizing multiple multiresolution convolutional residuals in dense block, effectively reducing the number of superresolution network parameters, and increasing model hierarchy and network depth, with prediction speed reaches 25 ms per image under certain accuracy loss. Single-branch convolutional residual block can only extract single-level semantic information; Wang et al. [44] designed multiscale residual network (EMRN) in image superresolution task and densely connected multiscale residual blocks extracted image hierarchical features to achieve multilevel semantic information in different perceptual domains.

Third, residual dense block is dense block using residual connection between inputs and outputs, and residual dense block inherits advantages of ResNet and DenseNet, improves information flow of network, and retains more structural information. Wu et al. [45] proposed dense pyramid residual network (DPRnet) for restoring fog-free images, residual dense block extracts network features and quadruple downsampling features, global residual connection is applied to whole network, allowing the feature maps to be heavily reused and deepening network at different scales, and ultimately, DPRnet has better performance.

Fourth, multibranch residual dense block is dense block that uses both residual connection and convolutional residual connection, and multibranch residual structure improves diversity of feature extraction for hierarchical feature extraction. Long et al. [46] proposed aggregated residual dense network (RXDNFuse), which fuses residuals and convolutional residuals from infrared and visible images into parallel dense blocks to extract hierarchical features, and qualitative evaluation showed that rich texture details and prominent thermal radiation information were effectively retained.

#### 3.3.3. Overall Structure of Dense Unit

DenseNet is connected with dense units in a dense connection, and overall structure of dense unit is replaced with whole structure; five overall structure improvements are as follows.

First, multirate extended convolutional structure MDCS replaces dense unit, 1 × 1 convolution, and four different extended rate 3 × 3 convolutions to perform input feature mapping in parallel, which was used in training DenseNet by Zhang et al. [47], using original image and marker point set, adding MDCS to increase network width laterally to fully utilize multiscale vascular spatial feature information at lower computational cost, and dense connection alleviates gradient disappearance and accelerates network, the average dice score reached 93.20% with good integrity and sensitivity for cerebral vessel segmentation, especially for slender vessels. Second, Wang et al. [48] proposed dual residual attention model (DRAM) for SISR task, in which dense units tandem two 3 × 3 convolutions capture more global information, channel attention features and convolutional layer features share information in tandem, spatial attention features and convolutional path features are fused, and dual feature fusion enables the model to capture important high-frequency components; the superiority of model in benchmark dataset was verified. Third, Zeng et al. [49] used densely connected cascade network DCCN for MRI reconstruction of *K*-space undersampled data, using dense unit tandem residual dense block (RDB) and data consistency layer (DC), with RDB combining dense connection and residual learning to fully utilize features of different layers, and DC used data consistency to improve MRI image reconstruction under different sampling trajectories and sampling rates. Fourth, Zhang et al. [50] used densely connected inception-Res module for segmentation of medical images, with branches of different kernel sizes aggregating feature maps, widening network to learn more features, and residual connection referencing input feature mapping learning function to make the module easier to learn; final model achieved better performance on lung and brain tumor segmentation datasets. Fifth, Qamar et al. [51] proposed encoder dense block with residual connection for skin lesion segmentation architecture, using residual connection to obtain more robust features, dense unit also uses expanded space pyramid pools (ASPP) to efficiently capture global multiscale contextual information while maintaining network parameters, ASPP also aggregates image space information, and the results of study showed that architecture achieved the most advanced performance.

### 3.4. Dense Connection Mode

**Figure 7:**
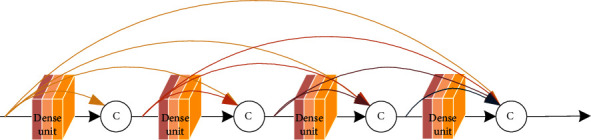
Dense connection mode.

Dense connection mode is two-by-two interconnection of all dense units in same dense block, and the feature maps of same scale are connected using channel stitching, as shown in Figure 7.

Dense connection mode [9] maximums information flow and reduces the parameters, while alleviating network gradient disappearance and overfitting phenomena, dense connection has following features: first, dense connection uses output as input to subsequent layers, enhancing feature transfer and using features more efficiently; second, dense unit in dense connection is narrower, adding only a small number of feature maps to network each time without relearning redundant feature maps, and final classifier makes predictions based on all features, maintaining network performance while reducing the number of parameters; third, each layer in dense connection directly uses gradient of loss function and initial input information, which is equivalent to invisible depth supervision and helps alleviate gradient disappearance; fourth, dense connection also has a regularization effect, which has a suppressive effect on overfitting, and network also can mitigate overfitting with fewer parameters. Dense connection improvement has four aspects: splicing method, connect dense unit of different scales, topological structure, and combine with other structure.

#### 3.4.1. Splicing Method

**Figure 8:**
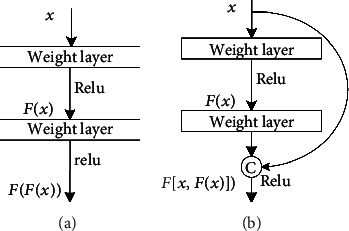
(a) Traditional CNN unit mode and (b) DenseNet unit mode. (a) Input gradient information in conventional unit module goes through many layers. (b) DenseNet unit module uses dense jump connection to splice input features with learned features as subsequent layer inputs thus achieving feature reuse, which is easy to train and more parameter efficient in DenseNet. Specific structural design details can be expressed in following equation with some notations defined in advance: input picture is *X*_0_, *l* is index of neural network layers, *H*(•) indicates nonlinear operations combination, and *H*(•) in DenseNet refers to composite function consisting of BN, ReLU, and Conv.

Current unit input in dense connection is stitched from previous unit feature maps, and stitching method is to perform channel stitching at same feature map size. Traditional convolutional neural network unit module is different from dense connection neural network unit module, as shown in Figure 8.

Output of the first layer of conventional neural network can be expressed as

(1) $$\begin{array}{c} X_{l}=H\left(X_{l-1}\right). \end{array}$$

**Figure 9:**
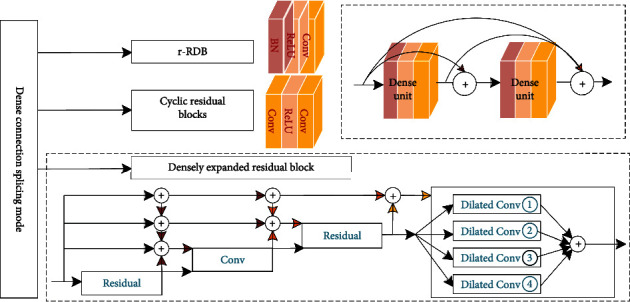
Dense connection splicing mode.

DenseNet splices different layer features, output of the first layer is expressed as:

(2) $$\begin{array}{c} X_{l}=H\left(\left[X_{0},X_{1},\cdots \cdots ,X_{l-1}\right]\right), \end{array}$$

where [*X*_0_, *X*_1_, ⋯⋯, *X*_*l*−1_] represents 0 ~ (*l* − 1) layer feature map splicing; dense connection mode is shown in Figure 9.

First is residual dense connection mode for channel fusion. Zhang et al. [52] proposed improved residual dense blocks (r-RDB) in image string and handwritten character recognition tasks, maintaining RDB local feature fusion and local residual learning capabilities, cascading multiple r-RDBs to form global dense block using dense connection of residual summation, and adaptively learning global dense residual features to ensure interblock information flow, r-RDB feature fusion, and residual learning refine block structure and reduce inner computational cost. Adegun and Viriri [53] proposed DenseNet framework for cyclic residual block classification based on dense block, where dense connection within cyclic residual block is combined using summation operations to reduce computational effort, contour refinement, and localization of lesion boundaries for edge potential; results showed that effective preprocessing and segmentation of skin lesions can improve classification performance.

Second, dense-expanded residual block improves dense connectivity as a method for multiplexed residual fusion, Hong et al. [54] proposed an end-to-end network MMCL-Net for simultaneous detection, segmentation, and classification of spinal structures, establishing spatial dynamic connectivity for three structures and three tasks, dense connectivity for multilevel aggregation of expanded residual unit to extract important radiological features, and dense connectivity as residual mapping; shared learning among multiple tasks represents adaptive optimization of model, and densely expanded residual module outputs are received in parallel by dilated convolutional layers with different expansion rates; MMCL has been validated on MR images of more than 200 clinical patients, and it can accurately identify structurally relevant lesions in spine and show superiority in grading lumbar spinal neural foraminal stenosis.

#### 3.4.2. Connect Dense Units of Different Scales

**Figure 10:**
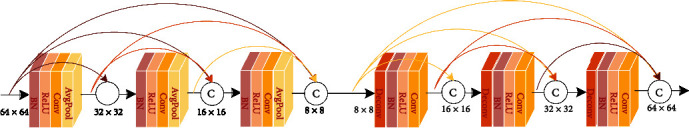
Connect dense units of different scales [55].

Dense connection in DenseNet is to connect same scale dense unit feature maps, and connecting dense units of different scale is shown in Figure 10.

Dense connection is used between different scale structures of encoders or decoders. Wang et al. [56] used dense connection within encoder and decoder networks, average pooling between convolutional layers to eliminate redundant features, and dense connectivity of units with different feature sizes to adequately extract biomarkers associated with recurrence of high-grade plasmacytic ovarian cancer from CT data; the model provides a new prognostic analysis approach. Pemasiri et al. [57] used multimodal semantic segmentation model to segment human body parts in visible and X-rays, where densely connected features were adjusted to dense units of same size, and features of different modalities were integrated using channel splicing to achieve single model for multimodal semantic segmentation. There is only one set of cascade layers between U-Net codec blocks, and Jun et al. [58] designed nested codec architecture (T-Net) with pooling and upsampling inserted in codec blocks, feature mappings interconnected, encoder low-level and high-level features connected to whole decoder, and feature mappings at different scales to produce complex semantic segmentation, and experiments showed that Dice similarity coefficient of T-Net reached 83.77%, which is 10.69% higher than that of U-Net.

#### 3.4.3. Topological Structure

**Figure 11:**
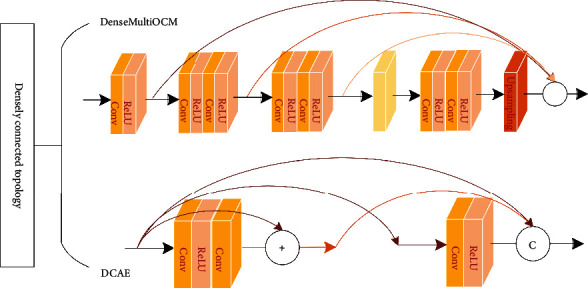
Densely topological structure.

Conventional *L*-layer convolutional neural network has *L* connections, while DenseNet has *L*(*L* + 1)/2 connections, densely connected *L*th layer has *L* inputs, which are feature maps of its predecessors, and its feature maps also need to be passed to all subsequent layers, as shown in Figure 11. Due to this two-by-two interconnected connection, DenseNet has constant mapping, deep supervision, and deep diversity characteristics, densely connected approach has a simple internal representation and can reduce redundant features.

First, dense connection topological structure is to aggregate outputs of all dense unit to the end of dense block. In superresolution (SR) domain, deeper network helps to improve image reconstruction quality and introduce residual connection to avoid gradient disappearance, but deep network is computationally intensive and inefficient, Yang et al. [59] used a new dense connection approach to extract low-resolution image features; model topology is to merge outputs of unit into the end of module and obtain deeper network and richer feature map by reducing the number of channels and network parameters; model was experimentally shown to be effective in SR with different magnifications. Mostefa et al. [60] proposed DenseMultiOCM for extracting MRI image features in brain tumor segmentation, improved dense connection by connecting only unit output to final output of dense block, dense units in series to two 3 × 3 convolutions, and final unit used pooling and upsampling to extract half-scale features; experiments showed model segmentation results were improved.

Second, residual connection is introduced in dense connection mode, two methods of SR are to learn features in high-resolution (HR) and low-resolution (LR) image space, and HR method is to interpolate LR information into HR, but it increases computational complexity and may introduce additional noise; LR method is to extract features and then increase spatial resolution by deconvolution, which is less computationally intensive, but difficult to learn multiscale nonlinear LR-HR mapping. Both methods learn mapping relationships in single-scale image space and cannot provide SR information across different scale of features, and although there is a Laplace pyramid structure to progressively upsample LR information to generate multiscale features, it also uses only highest-level features and ignore low-level features, Zhou et al. [61] designed dense convolutional autoencoding (DCAE) block to extract multispatial scale features with different spatial resolution and temporal scales to establish multilevel feature reuse mechanism, and DCAE units were stitched with dense concatenation and jump concatenation to achieve long-term temporal feature reuse, and benchmark evaluations showed that this method outperforms existing methods, especially for structured images.

#### 3.4.4. Combine with Other Structures

Dense connectivity method connects other structures with the following six densely connected structures.

First, Signoroni et al. [62] proposed BS-Net for predicting pneumonia in chest X-ray images, segmenting and predicting disease severity with dense connection to encode stage feature maps and also using densely connected deconvolution layers instead of code and decode jump connections, and model exceeded radiologists' scoring accuracy and consistency on the public COVID-19 dataset. Second, deep learning rarely focuses on both accuracy and speed in medical image segmentation and extends to mobile environments, where multiple downsampling loses significant information, Tseng et al. [63] densely connected U-nets at different scales to form dense blocks, dense connection improves feature reuse and preserves features at each step, and deconvolution enables upsampling to retain as much information as possible; DNetUnet achieves better segmentation performance and can be a mobile platform. Third, Liu et al. [64] designed DRN-DCMB model for reducing motion artifacts in brain MRI, where dense units of multiresolution block consist of U-net-like structure; four resolution features of U-net are convolved and then stitched together, where inputs and outputs of residual connectivity model predict image artifacts; learning residual mapping can significantly reduce motion artifacts and maintain image contrast, while improving image quality and sharpness. Fourth, Wang et al. [65] proposed multipath connectivity network (MCNet) for medical segmentation, where the encoder extracts different size features using multiscale feature blocks and densely connects same size feature maps in codec, and rich contextual information improves segmentation effect; MCNet has strong robustness and better performance for different scale targets. Fifth, for performance improvement, network structure improves from simple to complex, but simple stacking of capsule layers does not improve performance; too much stacking will lead to too small coupling coefficients and inhibit gradient, capsule network dynamic routing algorithm requires a lot of computation and storage costs, parameter increase will also lead to serious overfitting, so reasonable architecture is the key of deep capsule network, and lightweight of deep capsule network is also important research direction; Sun et al. [66] designed structure based on multilevel separable convolution and dense capsule layers, with smaller performance loss while effectively reducing parameters, dynamic road algorithm is lightweight with separable matrix, densely connected capsule layers increase network depth and complexity, competitive performance can be obtained with fewer parameters, and model is quantitatively demonstrated to have optimal parameter utilization through accuracy and parameters number ratios. Sixth, variation in image scale limits performance of target detection and semantic segmentation models to some extent, and crowd counting domain is prone to significant variation in crowd image size, leading to more obvious model performance limitations, Wang et al. [67] proposed single-column scale-invariant network (ScSiNet) for crowd counting with densely connected scale-invariant (SiT) layers forming dense block and SiT using different feature channel groups to encode different receiver domain images and fusing fine-grained multiscale information, using dense connection for multilayer feature interlayer fusion, and model combines intralayer scale aggregation with interlayer multiscale to obtain more accurate performance and computational precision with good metastability and scale invariance.

### 3.5. Attention Mechanism

Attention mechanism [68] is introduced into codec framework, which makes it easier to learn the intermapping relationships between multiple contents and different modal data; thus, allowing better representation and overcoming its uninterpretable and hard to design drawbacks, attention mechanism does not require supervised signals and appears to be extremely effective for problems with little a priori cognition. Attention module reduces computational burden of high-dimensional data by structurally selecting a subset of input and improves the quality of output by “eliminate false and retain true,” allowing model to focus more on useful information in input that is significantly relevant to current output. Introduce attention mechanism into dense unit, and DenseNet is based on three main considerations: first, allow model to achieve better performance in text image analysis tasks; second, attention mechanism can calculate weights for each input and improve the interpretability of network model operates way [69]; third, it can alleviate some defects in recurrent neural networks, such as performance degradation due to increasing sequence input length and computational inefficiency due to input ordering.

#### 3.5.1. Attention Mechanism in Dense Unit

**Figure 12:**
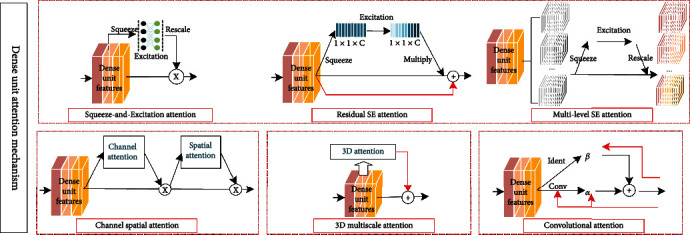
Attention mechanism in dense unit.

Dense unit introduces attention mechanisms and mainly squeezes excitation (SE), residual SE, multilevel SE, channel spatial, 3D multiscale, and convolutional attention mechanism, as shown in Figure 12.

First, SE attention mechanism is to increase skip connection of attention mechanism in adjacent layers, focuses on interchannel relationships and learns the importance of different channel features, and improves interchannel information interaction, thus improving network performance. Feng et al. [42] proposed densely connected recurrent network (DCRN) for single-image superresolution tasks, introducing SE for dense unit and dense block features, rescaling and extracting contextual information, and global pooling of SE attention mechanism to extract global structural information, and fully connected layer emphasizes information channels and suppresses useless channels to improve model representation and reconstructed image consistency. Huang e al. [55] proposed a preprocessing method to optimize diagnostic performance of meningioma or glioma and narrow intraclass gap, including multidirectional brain region extraction (MDBRE) method and bimodal iterative gamma correction, and combined SE and DenseNet classifier to extract brain regions; the accuracy of triple classification normalized luminance images was improved by 9.7%.

Second, residual SE attention mechanism is to add residual connection to SE branch. Shi et al. [70] combined dense connection with residual SE to propose myopic eye detection network (MDNet), which introduced residual SE correction for dense unit feature mapping channels, reduced the number of parameters, and improved network performance; network detected spherical equivalent mean absolute error up to 1.1150 d (Diopter); feasibility and applicability were verified in fundus images.

Third, multilevel SE attention mechanism is introduced after feature map grouping; personnel reidentification task is critical to capture appearance changes in different viewpoints; localization and alignment of body part are inaccurate, and it is difficult for CNN to capture distinguished information to generate robust representation of pedestrians, for which Yan et al. [71] based on interdependence between different channels and horizontal feature maps, using spatial attention mechanism to learn distinguished local features and construct multilevel SE attention blocks for different subnets, adaptively discover body parts and recalibrate features to solve misalignment problems caused by inaccurate pedestrian detectors.

Fourth, channel spatial attention mechanism is introduced in dense unit, and tandem channel attention and spatial attention constitute convolutional block attention module (CBAM), which can be widely used to improve convolutional neural network representations. Xue et al. [72] proposed dual-attention dense ATP-DenseNet for handwritten font recognition, in which CBAM channel attention aggregates spatial features of average and maximum pools; channel attention map is computed using shared multilayer perceptron; operations along channel axis can effectively highlight information regions; average and maximum pools process features to map channel features and generate a two-dimensional spatial attention map in tandem; CBAM is introduced into dense unit to facilitate interlayer information flow and refine features across layers, while target-scale features are given more attention.

Fifth, 3D multiscale attention mechanism is introduced in dense unit. Zhang et al. [73] used 3D densely connected convolutional neural network (CAM-CNN) to extract brain MRI multilevel features for classification of Alzheimer's disease and mild cognitive impairment, densely connected difference at different unit levels; 3D dense unit introduces attention mechanism to generate attention maps and sum transformed MRI hierarchical data into more compact high-level; model has high classification prediction accuracy, and classification performance is at the highest level.

Sixth, convolutional attention mechanism is introduced in dense unit, considers interlayer relationships, and makes full use of interlayer information in convolutional and skip connection. In superresolution tasks, the main difference between high-resolution and low-resolution images lies in edges and textures, which are mainly based on high-frequency feature information to guide image texture recovery, residual superresolution networks have similar convolution and skip processing, resulting in some high-frequency information being missed in network. Bai et al. [74] proposed dense convolutional attention networks (DCAN) to introduce convolutional attention mechanism, which introduces weighted skip connection in convolution, considering interdependence between layers and adaptively calibrating convolutional features to learn richer details and sharper edges by crossing layers; DCAN spends less time to obtain superior performance in both quantity and quality.

#### 3.5.2. Attention Mechanism in DenseNet

**Figure 13:**
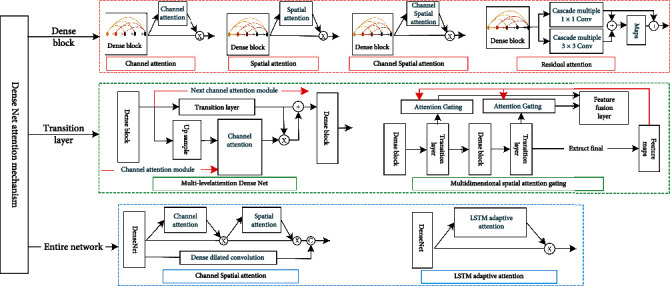
Attention mechanism in DenseNet.

DenseNet mainly consists of dense block and transition layer, introducing attention mechanism to enhance feature propagation and reduce the number of network parameters, and introducing attention mechanism in DenseNet is shown in Figure 13.

*(1) Attention Mechanism Introduced in Dense Block*. Attention mechanism introduced in dense block has channel, spatial, channel and spatial, and residual attention mechanisms.

First is attention mechanism in dense block for feature enhancement. Zhou et al. [75] proposed 3D SE-DenseNet for dynamically enhancing hepatocellular carcinoma grading in MR images, introducing SE to enhance key features while suppressing redundant features, feature maps are easier to fuse, and network obtains better performance and accuracy. Squeeze excitation simulates interdependencies between channels but has limited processing of contextual information, Wang et al. [76] used dilated dense unit to extract multiscale features for crowd counting, channel attention mechanism to guide dense block multiscale contextual feature fusion, adjustment of feature channel weights at each fusion stage, recalibrate of fused feature responses, and its convolution layer to capture channel correlation and global average pooling aggregated output feature spatial information; bootstrap channel fusion to adjust feature method outperforms other methods in population density map estimation.

Second, spatial attention mechanism is introduced in dense block. Wei et al. [77] proposed Att-DenseUnet for skin lesion segmentation; spatial attention module was added to encoding and decoding skip connection, using advanced semantic contextual information in encoding stage to focus on lesion region features and suppressing irrelevant artifact region features in dense block, and final model output had high shape accuracy, clear boundary, and high recall segmentation results; Zhang et al. [78] used a multiscale model to automatically classify traffic patterns and speeds, and multiscale spatio-temporal granularity grid data features were extracted in dense block; spatial attention map is obtained after element-by-element dot product of 1 × 1 convolution and sigmoid functions, which reduces the number of network parameters and enhances interchannel feature fusion and further enhances information flow in dense block using residual connection; final model outperforms existing models in terms of accuracy, recall, and *F*1 scores.

Third, channel spatial attention is introduced in dense block, which adaptively adjusts cross-channel and spatial region weight to improve network performance using interchannel and interfeature relationships. Qin and Gu [79] introduced channel and spatial attention to residual dense block and proposed deep multilevel residual attention network (MRAN) with enhanced image high-frequency features. Attention module aggregates channel information with feature statistics and multilayer perceptron compress channel information and sums it pixel by pixel, emphasizes information-rich channels, computes feature statistics to obtain spatial mapping, and multiplies element-by-element, and spatial attention maps along channel axis improve network perception, focusing more on informative rich features to recover more accurate details, and extensive evaluations show that MRAN is quantitative results and visual performance state-of-the-art methods.

Fourth, residual attention mechanism is introduced in dense block to maintain top-down spatial attention without interrupting bottom-up convolutional feature extraction process, and the residual connection attention mechanism fuses two different convolutional features and enhances feature representation. Bi et al. [80] proposed residual attention densely connected RADC for aerial image scene classification, residual attention mechanism highlights local semantics in output features, and experiments show that RADC with fewer parameters outperforms some existing methods.

*(2) Attention Mechanism Introduced in Transition Layer*. Attention mechanism introduced in transition layer has multilevel attention mechanism and multidimensional spatial attention mechanism with spatial attention gating.

First, multilevel attention mechanism is introduced in transition layer to fuse adjacent dense block output features and generate channel spatial attention map to enhance transition layer features. In diagnosing gastric cancer in gastric pathology section images, although DenseNet rich semantic information can detect larger sensory field data, it is still difficult to obtain spatial information and identify hidden gastric features in feature maps, Liu et al. [81] proposed multilevel attention dense network (MSA-DenseNet) to detect gastric cancer in 20x magnified section images; transition layer introduced attention providing attention vectors to enhance gastric features, selecting more semantic information about cancer, global average pool, and fully connected layer to provide nonlinearity for channel attention, using adjacent dense block features to enhance relevant semantic information, combining lower-order features with more spatial prediction and higher-order features with more semantic prediction features; final model obtains better detection results than existing manual detection methods.

Second, multidimensional attention with spatial attention gating is introduced between dense block at different scales to adjust multiscale feature fusion and improve network prediction capability. In noninvasive clinical image lung cancer diagnosis research, which lacks reliable extraction of fine-grained features in different imaging modalities, Qin [82] proposed multidimensional attention-based lung cancer classification architecture for PET and CT images, with 3D DenseNet processing different imaging modalities in parallel, embedding spatial attention gating in dense block, generating corresponding spatial mappings by high-level semantic features and SE, implementing multidimensional attention mechanism, enhancing fine-grained feature extraction, and effectively reducing feature noise; quantitative evaluation metrics and deep learning visualization analysis shows that architecture obtains 0.72 ROC curve area and 0.92 accuracy, which can effectively extract fine-grained features of different imaging modalities.

*(3) Attention Mechanism Introduced to Whole Network*. Attention mechanism introduced in whole network has channel spatial and LSTM adaptive attention mechanism. First, skin lesion regions vary in color, size, and shape; some light lesions are highly similar to normal skin, and boundaries of deeper lesion regions are complex, to segment model with sufficiently dense feature resolution, Ren et al. [83] used serial attention network (SANet) to segment skin lesion regions and introduced channel first followed by a spatial attention mechanism to aggregate global, local, and interchannel information using interdependencies between channel mappings to improve representation of semantic features and using spatial attention to select global contextual information and contextual relationships to make semantic features more compact and consistent; SANet captures interpixel and interchannel feature dependencies and achieves 0.7692 average Jaccard index in ISIC2017 dataset. Second, it is difficult for image caption generation tasks to correctly extract image global features and perform image regions for each word without ignoring partial words, Deng et al. [84] used DenseNet to extract features required for LSTM generation of sentences and introduced LSTM adaptive attention mechanism to improve correspondence problem of forced text and image regions, which was demonstrated on Flickr30k and COCO datasets showed that flexibility of caption generation was improved, with significant improvements in the BLEU and METEOR evaluation criteria.

## 4. Application of DenseNet in Medical Image Analysis

Reviewing the past 10 years, deep learning has seen a proliferation of improvements in models and algorithms, and availability and computational power of large-scale image data has continued to improve, solving many challenges in computer vision. In 2022, Li et al. [85] provided a comprehensive summary of deep learning research in medical image classification, detection and segmentation, alignment, and retrieval; applications of DenseNet for medical images mainly include image classification, segmentation, detection, alignment, reconstruction, retrieval, generation, enhancement, and fusion, but research and improvement of its technology mainly focus on classification and recognition, segmentation, and localization detection; medical image classification commonly used application scenarios are disease diagnosis; medical image segmentation projects are mostly applied to segmentation of lesions and organs, such as brain segmentation, lung segmentation, heart segmentation, and liver segmentation. Therefore, this section summarizes applications of DenseNet in field of medical image analysis from these three aspects.

### 4.1. Application of DenseNet in Medical Pattern Recognition

**Figure 14:**
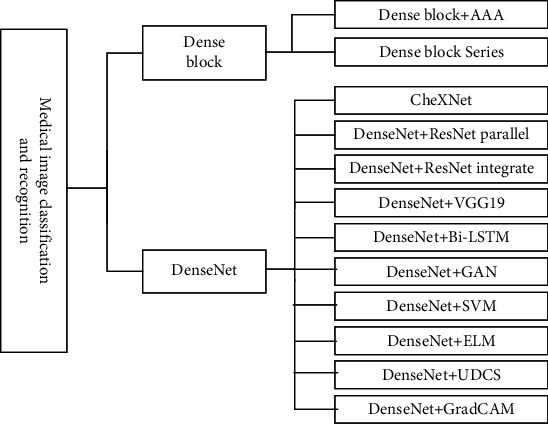
Application of DenseNet in medical classification and recognition.

Image pattern recognition is a popular research area in computer vision, which usually includes two steps: feature extraction and classifier. Medical image classification is further divided into image screening and lesion classification. Image screening uses detection images as model input to predict whether the disease is suffered or graded in severity. Lesion classification assists clinicians in diagnosing human lesions and grading the severity quantitatively, and it is important to identify tumors and nodules in human tissues and organs. This section summarizes applications of medical image pattern recognition from perspective of combining DenseNet with other methods; 12 improved methods are shown in Figure 14.

### 4.2. Combine Dense Block with Other Methods

First, Guo and Yuan [86] combined dense block and adaptive aggregation attention (AAA) module to classify wireless capsule endoscopy images (WCE); AAA captured global correlation to estimate abnormal regions and obtained 93.17% accuracy in quadruple crossover on CAD-CAP WCE dataset. Second, in pediatric radiology and forensic medicine, bone age assessment is one of clinical diagnostic techniques to evaluate skeletal development in children, Guo et al. [87] tandem six dense blocks to perform comprehensive evaluation of high- and low-quality X-ray images to achieve state-of-the-art performance under MAE metric.

### 4.3. DenseNet Variants

Rajpurkar et al. [88] proposed 121-layer convolutional neural network CheXNet based on DenseNet, applied to the largest publicly available chest X-ray dataset ChestX-ray14, which contains over 100,000 X-ray images of 14 diseases, and CheXNet exceeded average radiologist *F*1 metrics on a test set annotated by four practicing radiologists performance.

### 4.4. Combine DenseNet with Others

DenseNet integrates with other networks: first, chest X-ray images, as the most common screening technique for diagnosing chest diseases (atelectasis, cardiomegaly, pneumonia, etc.) suffer from complex background, many potential abnormalities, diverse interactions between abnormal patterns, etc. Traditional techniques focus on low-level features such as appearance, texture, and contrast, although deep networks can extract higher-level features, single network in intermediate layers also loses some unique details, and homogeneous networks learning complementary features from each other may overlap, Chen et al. [89] used DualCheXNet, a dual asymmetric network, to improve performance of multilabel chest disease classification, with parallel asymmetric subnetworks DenseNet and ResNet both using jump connections to create short paths, and enhanced gradient propagation from previous to following layers, residual connection to reuse early features, and dense connection to mine new features; advantages of different unique feature extraction methods complemented each other and were validated on ChestX-ray14 dataset, obtaining average AUC score of 0.823. Second, Srivastva et al. [90] proposed PlexNet integrated with pretrained DenseNet and ResNet for ECG biometrics, feature level fusion approach for tandem features, and fully connected layer based on similarity classification, using both migration learning and integrated learning to take advantage of model effectiveness and robustness demonstrated on PTB and CYBHI datasets. Third, Kedia et al. [91] combined DenseNet121 and VGG19 into CoVNet-19 two-level stacking model, both models extracted chest X-ray features and minimized noise, SVM achieved three-level classification of COVID-19, pneumonia, and normal, obtaining 98.28% overall accuracy and 97.15% Mathews correlation coefficient and *F*1 scores of 99% in both binary classifications.

DenseNet combined with other network structures; fourth, Li et al. [92] combined DenseNet and bidirectional recurrent neural network (Bi-LSTM) to predict vivo RNA transcripts from in vitro data; Bi-LSTM learns RNA sequences, secondary structure information and pairwise probability features, which were reshaped into matrices and fed into DenseNet with prediction accuracy and scalability in with better performance. Fifth, Man et al. [93] screened mislabeled plaques in breast histopathology images based on generative adversarial network (GAN) unsupervised abnormality detection; DenseNet extracted multilevel features of plaques for benign and malignant classification, achieving excellent results on coarse-grained high-resolution images especially 40x and 100x images.

DenseNet is combined with other methods; sixth, Devnath et al. [94] used CheXNet to extract multilevel features of chest X-ray images, mixed SVM and CNN aggregation methods, transition layer low-level features, and deep-level features mapped to high-dimensional space after merging dichotomous classification. Seventh, Turkoglu [95] proposed a multicore extreme learning machine (ELM) method to extract deep-level features of chest CT with pretrained DenseNet201, and ELM classifier classified features with different activation methods to predict final presence of neo-coronary pneumonia by majority voting. Eighth, Yuan et al. [96] addressed the problem of imbalance and discrepancy between small and large categories in wireless capsule endoscopy images and proposed DenseNet-UDCS polyp recognition model by calculating suitable features, unbalance discriminant loss (UD) function to improve model sensitivity to minority groups and category-sensitive loss (CS) constraint to learn features in hierarchically between different categories, so that intraclass differences minimized and interclass distance within batches maintained, with more advantages in model recognition accuracy, computational speed, and stability. Ninth, Ellis et al. [97] mixed weakly supervised, extrasupervised, and annotated classification of two-view chest radiographs; DenseNet-121 extracted features to generate 1D global feature vector and estimated scores; fully connected layer gradients were convolved with feature sets to estimate gradient class activation maps (GradCAM); attention mining branch learned GradCAM threshold operations using DenseNet-121, which masked important classification regions in input, increasing accuracy and sensitivity to negative prediction values, despite a decrease in false positives and decreases in overall accuracy.

### 4.5. Application of DenseNet in Medical Image Segmentation

**Figure 15:**
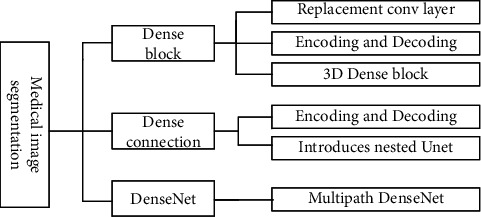
Application of DenseNet in medical image segmentation.

Medical image segmentation is to identify contour of target region or internal voxel collections, which is a key task for clinical surgical images to guide tumor radiotherapy, medical image organ and lesion segmentation allows quantitative analysis of volume and shape-related clinical parameters. Effective automatic organ segmentation is very challenging, medical images have high complexity, lack of simple linear features, insignificant brightness differences between organs, connected to surrounding organs, and blurred boundaries, as well as interference factors such as image artifacts and noise, and segmentation results accuracy is also affected by partial volume effects, gray scale inhomogeneity, artifacts and gray scale proximity between different soft tissues. Traditional segmentation algorithms are difficult to process images accurately, and application of DenseNet makes medical image segmentation effect improved, summarizing the seven models shown in Figure 15.

#### 4.5.1. Dense Block in Medical Image Segmentation Application Research

First, dense block replaces convolutional layer. Ding et al. [98] used dense block instead of codec convolutional layer to extract low-level visual features of multimodal brain tumors, and fused high-level semantic features to generate high-resolution features with fewer network parameters and fast segmentation, achieving good segmentation results. AIGhamdi et al. [99] introduced dense block in U-net for breast artery calcification detection; DU-net has dense connectivity, which helps to improve computational reusability and gradient mobility and improves accuracy and training difficulty. Second, codec introduces dense block. Vila et al. [100] performed semantic segmentation of carotid ultrasound plaques based on DenseNet, dense connectivity captured multiscale contextual information, and correlation coefficient of carotid intima-media thickness reached 0.81 in the experiment; robustness and generalization ability were validated on two datasets. Thirdly, 3D dense block was applied to segmentation networks. Bui et al. [101] proposed 3D-SkipDenseSeg, an accurate segmentation method for infant brain MRI, in which jump connections directly combine fine and coarse feature information from different levels of dense block to alleviate low accuracy problem caused by low-intensity contrast between MRI tissues, final model obtained the best dice similarity coefficient of 90.37, and segmentation accuracy and parameter efficiency are high.

*(1) Dense Connection in Medical Image Segmentation Application Research*. First, dense connection is introduced in codecs. Shi et al. [102] proposed dual dense context-aware network (DDCNet) to automatically segment hippocampus; dense connection is introduced between codecs to fuse high level and low level multiresolution features and contextual information, which can enhance interlayer feature propagation and improve information flow between codecs. Second, nested U-net introduces dense connection; Li et al. [103] proposed ANU-Net combining deeply supervised codec structure and nested segmentation network, designed full resolution nested U-net between codecs, introduced dense connection between nested convolutional layers of same resolution to obtain full-resolution features at different semantic levels, and experimentally demonstrated that ANU-Net can suppress irrelevant background regions while increase target region weight.

*(2) DenseNet in Medical Image Segmentation Application Research*. Multipath DenseNet extracts features. Kitrungrotsakul et al. [104] proposed multipath DenseNet for hepatic vascular segmentation, binary classification of blocks in three planes, sagittal, coronal and transverse; IRCAD dataset experiments show that the model is currently the latest method accuracy.

### 4.6. Application of DenseNet in Medical Object Detection

Localization of anatomical structures and lesion regions in human organs is an important preprocessing step in clinical treatment planning and intervention, and localization of specific biomarkers or anatomical structures in medical images is directly related to treatment outcome. The key target of interest or lesion detection is to classify each pixel; DenseNet is applied to improve the effectiveness of medical image localization detection, with the following three main models.

First, Ghatwary et al. [105] detected esophageal abnormalities in endoscopic images based on faster R-CNN- and DenseNet-enhanced feature propagation between layers, combined with Gabor manual feature enhancement to detect texture details in stage, obtaining 92.1% and 91% accuracy on MICCAI2015 and Kvasir datasets. Second, Liu et al. [106] proposed fully convolutional dense pixel classifier (FC-DenseNet) depicting ribs and clavicles, aggregating contextual information with jump connections, dense blocks instead of upsampling paths in convolutional layer, and dense connections to enhance feature propagation and reuse, and FC-DenseNet automatically depicts chest radiographs from multiple public databases to generate accurate binary edge maps, verifying the effectiveness of the model for bone suppression. Third, Xiao et al. [107] designed Siam network based on DenseNet to track vessel wall displacement from ultrasound RF signals and target blocks of labeled data as convolutional kernels to calculate blocks in search region with similarity score maps, achieving first single-target displacement tracking in ultrasound pulse wave imaging, accurately predicting local mechanical properties of arteries, and tracking better than traditional single-target block matching better.

## 5. Summary and Outlook

In summary, DenseNet, which can learn deeper and more discriminative features from images, has been applied to several research areas of medical image analysis and has made breakthroughs; although DenseNet has been effectively validated in publicly available and relevant medical image databases [41], it still faces many challenges. First, the huge amount of medical data outside images [4] contains rich information, but it is difficult to be fully utilized, such as additional labels indicating lesion conditions, clinical diagnosis reports reflecting physicians' conclusions and descriptions, which can help deep learning models to improve diagnostic performance, and recurrent convolutional neural networks (RNN) can be utilized to incorporate medical other data into DenseNet.

Second, deep learning models rely on a large number of manually labeled datasets [108, 109], which usually require significant effort and time to collect, clean, and debug data, and changes and evolution of actual task requirements also lead to retagging of the datasets, data dependence of models and cost of dataset labeling are issues that need to be urgently addressed by researchers. With the limited labeled trainable data [110, 111] currently available for training, semisupervised and weakly supervised methods can be used to learn unlabeled, weak, and small portions of labeled data.

Finally, there are still many challenges to training a model with higher classification or prediction accuracy and better generalization ability. There are limitations in using multiple branches to achieving DenseNet broaden; DenseNet lightweight is not only studied from the direction of architecture design and model compression but also based on data considerations, such as dimensionality reduction of intermediate features; dense connections lead to redundancy of feature extraction in different layers; dense block has excellent performance, but transition layer structure of dimensionality reduction is too simple, and it is difficult for single sensory field to capture multilevel features of dense block; the existing attention mechanism is basically in form of “weighting,” and appropriate attention mechanism should be designed according to data characteristics, such as residual shrinkage network [112]; “soft thresholding” is used to cope with strong noisy data.

Massive medical domain data presents challenges and opportunities for researchers in medical image analysis, by appropriate integration methods, different types of domain knowledge are used to assist deep learning to better accomplish corresponding tasks, and natural image pretraining weights are also introduced to improve network performance in medical domain. The massive data in medical image analysis will provide inexhaustible power as network architecture and algorithms improve; dense block and dense connection in DenseNet can be optimized for these new models, and DenseNet-base derivative models may also yield significant improvements in results for specific tasks.

## Data Availability

The data used to support the findings of this study are available from the corresponding author upon request.
